# Model Test of Strip Footing Behavior on Embankment Reinforced with Geogrid with Strengthened Nodes Under Static and Dynamic Loadings

**DOI:** 10.3390/polym17172331

**Published:** 2025-08-28

**Authors:** Chengchun Qiu, Zhuyi Xu, Dan Zhang, Mengxi Zhang

**Affiliations:** 1 School of Civil Engineering, Yancheng Institute of Technology, Yancheng 224051, China; xzy2210825408@163.com (Z.X.); feiguohai_2010@126.com (D.Z.); 2 Department of Civil Engineering, Shanghai University, Shanghai 200072, China; mxzhang@i.shu.edu.cn

**Keywords:** geogrid, embankment, strip footing, dynamic loading, bearing capacity, lateral displacement

## Abstract

The rapid development of transportation infrastructure in mountainous terrains, soft-soil foundations, and high-fill embankments poses stability challenges for conventional embankments, driving the application of advanced three-dimensional reinforced soil technologies. Geogrid with Strengthened Nodes (GSN) is one such innovation, forming a three-dimensional structure by placing block-shaped nodes at geogrid rib intersections. Current research on GSN focuses mainly on pullout tests and numerical simulations, while model-scale studies of its load-bearing deformation behavior and soil pressure distribution remain scarce. This study presents laboratory model tests to assess the reinforcement performance of GSN-reinforced embankments under static and dynamic strip loads. Under static loading, the ultimate bearing capacity of GSN-reinforced embankments increased by 74.58% compared with unreinforced cases and by 26.2% compared with conventional geogrids. Under dynamic loading, cumulative settlement decreased by 32.82%, and lateral displacement at the slope crest was reduced by 64.34%. The strengthened node design improved soil shear strength and controlled lateral deformation via enhanced lateral resistance, creating a more stable “reinforced zone” that alleviated local stress concentrations. Overall, GSN significantly enhanced embankment bearing capacity and stability, outperforming traditional geogrid reinforcement under both static and dynamic conditions, and providing a promising solution for challenging geotechnical environments.

## 1. Introduction

As transportation infrastructure rapidly advances, especially in mountainous regions, soft ground foundations, high-fill embankments, and constrained construction sites present significant technical obstacles for traditional embankment slope designs [1]. For example, in complex terrain and soil conditions, embankment slopes often exhibit insufficient bearing capacity, excessive settlement deformation, and inadequate stability, which seriously affect the safety and durability of the project [2]. Reinforced earth technology has been developed and widely applied to address these issues as an effective engineering measure [3,4].

The core idea of reinforced earth technology is to improve the mechanical properties of soil by arranging reinforcement materials within it [5,6]. Among these, planar reinforcement techniques (e.g., geogrids, geotextiles) have been widely used in embankments [7,8] and slope engineering [9,10] due to their ease of construction, low cost, good adaptability, and seismic performance [11,12]. Numerous studies have investigated the bearing capacity, settlement characteristics, and displacement behavior of planar reinforcement materials used to reinforce slopes, including model tests [13,14], centrifuge tests [15,16], full-scale tests [17], and numerical simulations [18,19]. These studies have shown that planar reinforcement techniques can effectively enhance soil shear strength, limit lateral deformation, and significantly improve the stability of slopes. For instance, Liu et al. [15] conducted centrifuge tests, which showed that geogrid reinforcement had a reinforcing effect on the soil and enhanced the integrity of the embankment, thereby improving slope stability and reducing the horizontal lateral displacement of the slope and the settlement of the slope crest. The results indicated that the geogrid arrangement, number of layers, and reinforcement layer spacing significantly influenced the reinforcement performance of a geogrid-reinforced structure. Keskin and Laman [18] discovered that, depending on the geogrid arrangement, the ultimate bearing capacity can be increased by up to approximately four times compared to an unreinforced slope. Additionally, the transfer of footing loads to greater depths through the geogrid layers and the interlock between the geogrid and the sand contribute to reducing lateral and vertical displacements beneath the footing. However, existing studies primarily focus on the performance of geogrid-reinforced slopes under static loading, while embankment slopes often experience long-term dynamic loads. Therefore, a deeper investigation into their dynamic response characteristics is warranted.

The limitation of planar reinforcement is that its effectiveness primarily relies on the planar interaction of the reinforcement material, making it difficult to leverage the synergistic effects in a three-dimensional space fully. To enhance the reinforcement effect further, three-dimensional reinforcement technology has gradually gained attention from academia and industry. The core of three-dimensional reinforcement technology is in forming a three-dimensional structure to improve the integrity and coordination between reinforcement layers. For example, Zhang et al. [20] proposed a 3D reinforcement system, demonstrating that vertical reinforcement elements could provide additional lateral resistance, significantly improving the reinforcement effect. Based on this, horizontal–vertical inclusions [21,22,23] and horizontal–vertical geogrids [24,25] have been developed. Additionally, technologies such as bearing reinforcement [26,27,28] proposed by Horpibulsuk et al., grid–anchor reinforcement systems [29,30] studied by Mosallanezhad et al., and stereoscopic geogrids with thickened transverse ribs [31,32] have all verified the significant advantages of three-dimensional reinforcement over planar reinforcement.

Among the research on three-dimensional reinforcement technology, geogrid with strengthened nodes (GSN) has attracted considerable attention due to its unique structural design. GSN forms a spatial mesh structure by arranging block-shaped strengthened nodes at the cross-points of geogrids (or by making the nodes more prominent) [33,34,35]. This design enhances the lateral resistance effect of the nodes, thereby improving the interlocking effect between the geogrid and the soil and demonstrating superior performance. Zhang et al. [36] conducted pullout tests and found that strengthened nodes considerably increased the pullout resistance of geogrids, subsequently developing a theoretical model for the pullout force based on the puncture–shear failure mechanism. Additionally, they observed that when nodes had uniform thickness, a double-sided arrangement was more effective than a single-sided one on the upper side, a conclusion that was corroborated by Du et al. [35] through discrete element analysis.

Currently, research on GSN is primarily focused on pullout tests and their corresponding theoretical and numerical analyses. However, there is a lack of research on the bearing performance and deformation characteristics of GSN when applied to embankment reinforcement under static and dynamic loads. This study focuses on GSN-reinforced embankments and investigates their performance under static and dynamic loads through laboratory model tests. The specific research contents include the ultimate bearing capacity under static loads, cumulative settlement and slope displacement under static and dynamic loads, and soil pressure characteristics. A comparative analysis with traditional geogrid reinforcement was also conducted.

## 2. Experimental Materials and Methods

### 2.1. Experimental Materials

#### 2.1.1. Sand Sample

**Figure 1:**
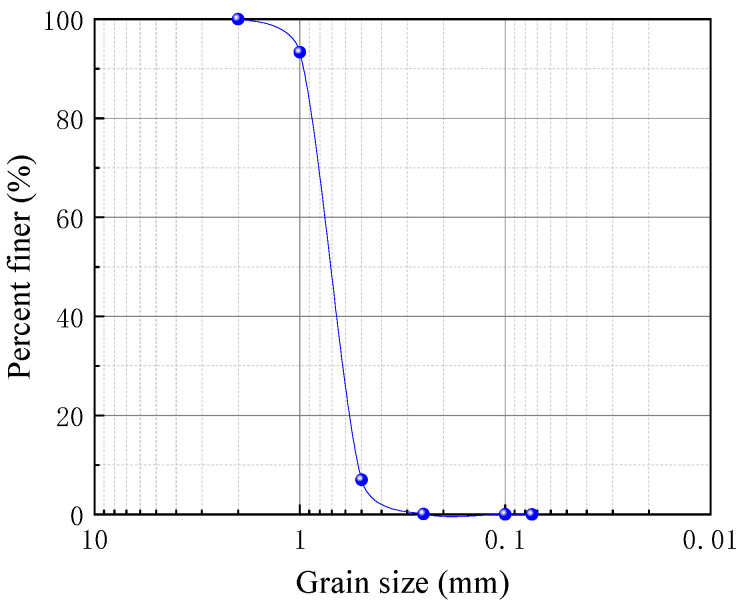
Grain size distribution curve of test sand.

The test fill material used in the model tests was local sand. The sand was dried at 105 °C and subjected to direct shear tests under a relative density of 0.7, yielding an internal friction angle of 33.7°. After basic tests, the particle size range was determined to be 0.5–2 mm, and the gradation curve of the test sand is shown in Figure 1. The sand is classified as poorly graded. Other physical properties are listed in Table 1.

#### 2.1.2. Reinforcement Material

**Figure 2:**
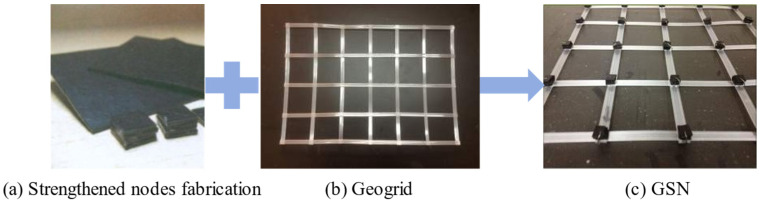
Geogrid with strengthened nodes.

The reinforcement material used was a biaxial polyester welded geogrid made of high-quality PET (polyethylene terephthalate), produced by Hubei Lite Geosynthetics Co., Ltd., Yichang, China, with an aperture size of 50 mm × 50 mm. Briefly, 6 mm thick blocks were fixed at the nodes to strengthen the geogrid nodes. The blocks were made of 1.5 mm thick high-density polyethylene geomembrane. The specific parameters of the geogrid and geomembrane are shown in Table 2 and Table 3, respectively. First, the geomembrane was cut into 6 mm × 6 mm pieces, then overlapped and glued with strong adhesive to form 6 mm thick blocks fixed to the nodes with steel wire, thus creating the GSN (Figure 2).

### 2.2. Experimental Apparatus

The internal dimensions of the model box were 600 mm × 290 mm × 400 mm (length × width × height). The frame of the model box was welded from 25 mm thick steel plates, while the sidewalls were made of high-strength transparent tempered glass to ensure rigidity and prevent deformation during loading. The transparent sidewalls facilitated the construction of the embankment model and allowed for image recording using a camera system. The left sidewall of the model box was detachable to facilitate the loading and unloading of fill materials. At the left end of the model box, there were two square columns with a rotating and movable circular rod in the middle, on which screws could be fixed. A hole was provided on the circular rod for mounting a displacement sensor, and the displacement sensor was secured with a screw to measure the distance between the rod and the slope surface.

The loading system for the model tests utilized a dynamic triaxial apparatus, with pneumatic loading and a maximum axial force of 10 kN and a maximum frequency of 5 Hz. The displacement sensor had a maximum range of 50 mm, and the pneumatic cylinder connected to the beam was installed on the two vertical columns of the model box. During the tests, the loading process was controlled by axial force.

### 2.3. Sample Preparation and Testing Procedures

**Figure 3:**
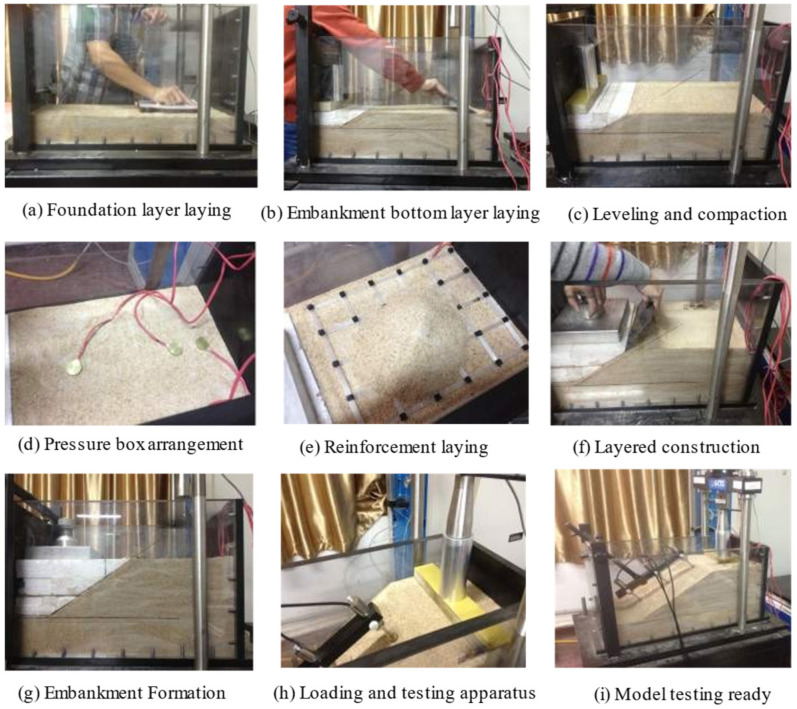
Model embankment construction flowchart.

**Figure 4:**
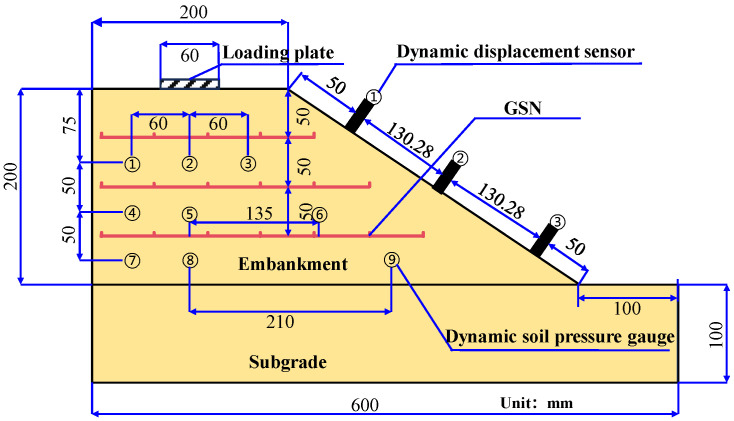
Dimensions of embankment model and layout of testing equipment.

The embankment model simulated a planned secondary highway project. The simulated embankment was 8 m wide, 6 m high, with a slope ratio of 1:1.5. Since the embankment was symmetrical, only half of the structure was modeled, with a model-to-prototype scale ratio of 1:20. The experimental model scale was determined as 1:20 due to the constraints of the laboratory equipment, specifically the dynamic loading system adapted from a dynamic triaxial apparatus. The model box dimensions were limited in size, but the resulting model (600 mm × 290 mm × 300 mm) aligns with established practices in similar studies. A comparison of model dimensions from previous studies is provided in Table 4. In this study, the ratio of model length to strip footing width is 10, and the ratio of model height to strip footing width is 5, both of which are close to the ratios reported in the literature. Based on a relative density of 0.7, the mass of the sand was calculated. First, a 100 mm thick layer of sand was laid at the bottom of the model box and compacted to simulate the foundation layer. The embankment was built in three layers, with three dynamic pressure cells installed in each layer. After each layer was laid, the material was compacted and leveled before the reinforcement material was placed on the surface. This process was repeated until the embankment reached the required height. Finally, dynamic displacement sensors were installed on the slope surface, and a loading plate with a width of 60 mm was placed at the center of the top of the slope to simulate a strip load in preparation for the loading process. Figure 3 shows the process of constructing a model embankment. The dimensions of the embankment model and the arrangement of the test devices are detailed in Figure 4.

The static load test was conducted using a stress-controlled continuous loading process. The load was increased at a constant rate of 4 N/s to ensure the jack remained vertical. The test was stopped when a sudden settlement occurred, indicating the ultimate bearing capacity had been reached.

The dynamic load test used a sinusoidal cyclic load to simulate traffic loads, with a frequency of 1 Hz and an amplitude of 60% of the ultimate bearing capacity of the unreinforced embankment, i.e., 33.3 kPa. The number of cycles was 20,000. The test conditions are listed in Table 5.

### 2.4. Experimental Limitations

The model was not strictly scaled according to similarity ratios; the geogrid and soil used were actual materials. Therefore, due to scale effects, the behavior of the granular soil in the laboratory model may differ from its behavior in the prototype. The preparation of the GSN was constrained by the limitations of small-scale model fabrication. Due to the difficulty of using small-mesh materials for creating strengthened nodes, actual geogrids with lower strength were used to form the GSN. This approach allowed for the qualitative investigation of the static and dynamic response of the slope reinforced with GSN. The method does not fully account for the strength and mesh-size reduction of the actual geogrid. To address this limitation, future studies will utilize 3D printing technology for the integrated fabrication of GSN with controlled polymer material properties. Validation should be conducted through large-scale model tests. However, since GSN is a new reinforcement form, preliminary tests are needed to demonstrate its advantages over traditional geogrids. The primary aim of this study was to observe the reinforcement effects of GSN-reinforced embankment models, providing useful references for future centrifuge tests or full-scale models.

## 3. Results and Discussion

### 3.1. Static Load Test

#### 3.1.1. Bearing Capacity

**Figure 5:**
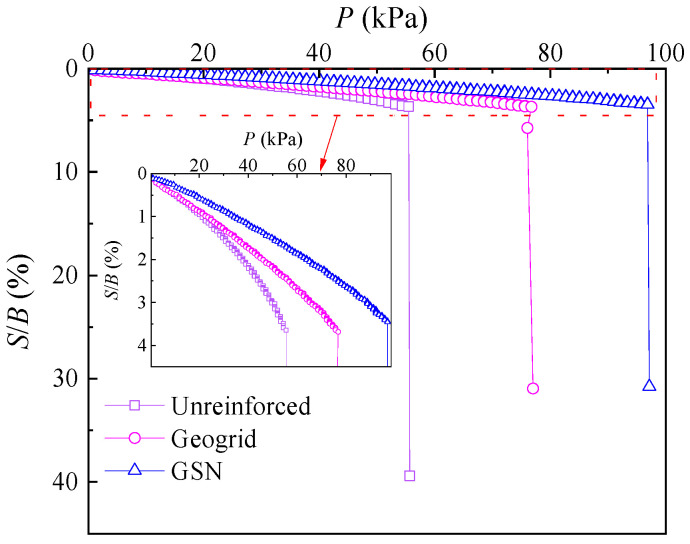
Load–settlement ratio curves.

During the test, it was observed that both unreinforced and reinforced embankments exhibited increasing settlement with increasing pressure until a certain stress value was reached, after which sudden settlement occurred. The load–settlement curve showed a steep drop, indicating the ultimate bearing capacity had been reached. The relationship between load (*P*) and settlement ratio (*S*/*B*, where *S* is settlement and *B* is the footing width) for various test conditions is shown in Figure 5.

As shown in Figure 5, the ultimate bearing capacity of the unreinforced embankment was 55.5 kPa. In comparison, the reinforced embankment with traditional geogrids had an ultimate bearing capacity of 76.77 kPa, an increase of 38.3% compared to the unreinforced embankment. The GSN-reinforced embankment had an ultimate bearing capacity of 96.89 kPa, a rise of 74.58% over the unreinforced embankment and 26.2% over the geogrid-reinforced embankment. The strengthened nodes in GSN significantly enhanced the reinforcement effect compared to traditional geogrids. The block-shaped strengthened nodes at the intersections of the geogrid significantly enhance the lateral resistance, which improves the interaction between the geogrid and the surrounding soil [33,35,36], leading to better interlocking and more effective load transfer. The enhanced lateral resistance also effectively limits lateral displacement, ensuring improved stability under both static and dynamic loads. Additionally, these nodes provide additional anchorage points and increase the overall stiffness of the reinforced soil mass, enhancing the soil’s shear strength and contributing to a higher ultimate bearing capacity.

Under a load of 40 kPa, the settlement ratio of the unreinforced embankment was 2.14%. In contrast, the reinforced embankment with geogrids had a settlement ratio of 1.71%, a reduction of 20.09% compared to the unreinforced embankment. The GSN-reinforced embankment had a settlement ratio of 1.17%, a decrease of 31.5% compared to the geogrid-reinforced embankment and 45.32% compared to the unreinforced embankment. The GSN effectively limited the settlement at the top of the embankment. Under a settlement ratio of 2%, the bearing capacities of the unreinforced, geogrid-reinforced, and GSN-reinforced embankments were 37.82 kPa, 46.07 kPa, and 63.86 kPa, respectively. The bearing capacity of the geogrid-reinforced embankment increased by 21.81% compared to the unreinforced embankment, while the GSN-reinforced embankment increased by 68.85% compared to the unreinforced embankment.

#### 3.1.2. Lateral Displacement Characteristics

**Figure 6:**
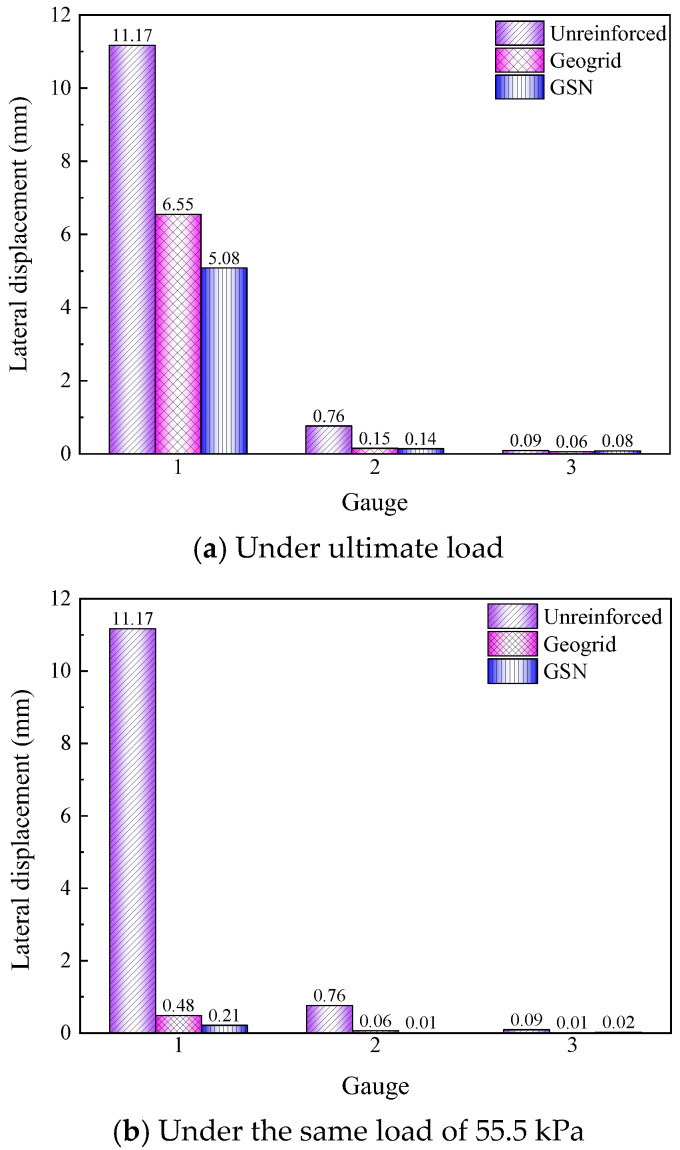
Lateral displacement of different cases under static loading.

The lateral displacement values at three measurement points under the ultimate load for the three test conditions are shown in Figure 6a. The lateral displacement decreased from the top of the slope to the toe, with the largest displacement occurring at the top and nearly negligible displacement at the toe. At the top measurement point (Point 1), the lateral displacement of the unreinforced embankment was 11.17 mm. The geogrid-reinforced embankment had a displacement of 6.55 mm, a reduction of 41.36% compared to the unreinforced embankment. The GSN-reinforced embankment had a lateral displacement of 5.08 mm, a reduction of 22.44% compared to the geogrid-reinforced embankment, demonstrating a significant reduction in lateral displacement.

For comparison, Figure 6b shows the lateral displacement values of the geogrid- and GSN-reinforced embankments at a load of 55.5 kPa, the ultimate bearing capacity of the unreinforced embankment. Under the same load, the geogrid effectively limited the lateral displacement of the slope, especially at the top, with a displacement of 0.48 mm, a reduction of 95.70% compared to the unreinforced embankment. The GSN-reinforced embankment had an even more significant reduction, with a displacement of 0.21 mm, a decrease of 98.12% compared to the unreinforced embankment. It was due to the frictional and interlocking effects of the geogrid and the additional lateral resistance provided by the strengthened nodes, which restricted the movement of the soil.

### 3.2. Dynamic Load Test

#### 3.2.1. Cumulative Settlement

**Figure 7:**
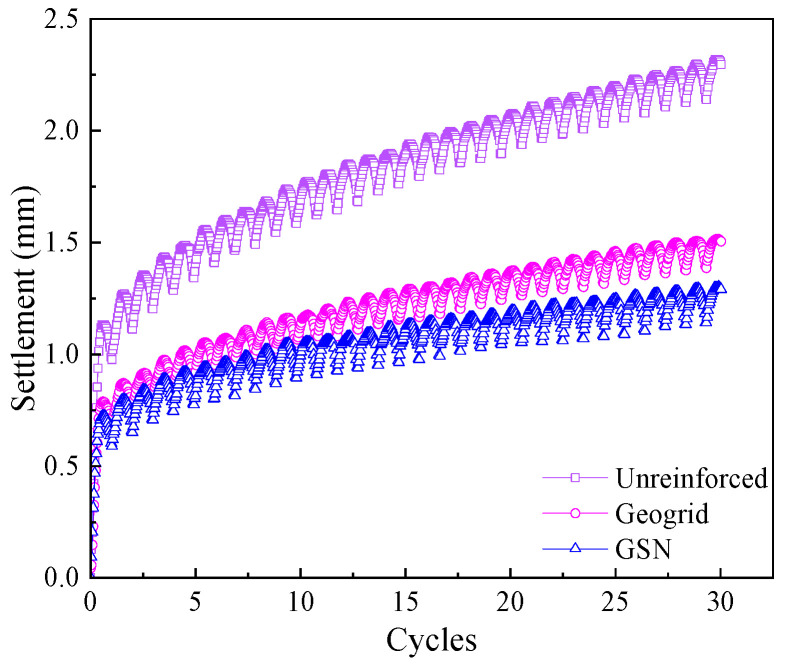
The settlement values over the first 30 cycles under different cases.

The dynamic load amplitude was set at 60% of the ultimate bearing capacity of the unreinforced embankment (55 kPa), i.e., 33.3 kPa, with a frequency of 1 Hz, a semi-sinusoidal waveform, and 20,000 cycles. Figure 7 shows the settlement–time curves for the unreinforced, geogrid-reinforced, and GSN-reinforced embankments in the first 30 cycles. Initially, the settlement values for all three conditions increased linearly, indicating soil compaction. As the number of cycles increased, the settlement values gradually increased in a semi-sinusoidal pattern, and the reinforcement effects began to take effect.

**Figure 8:**
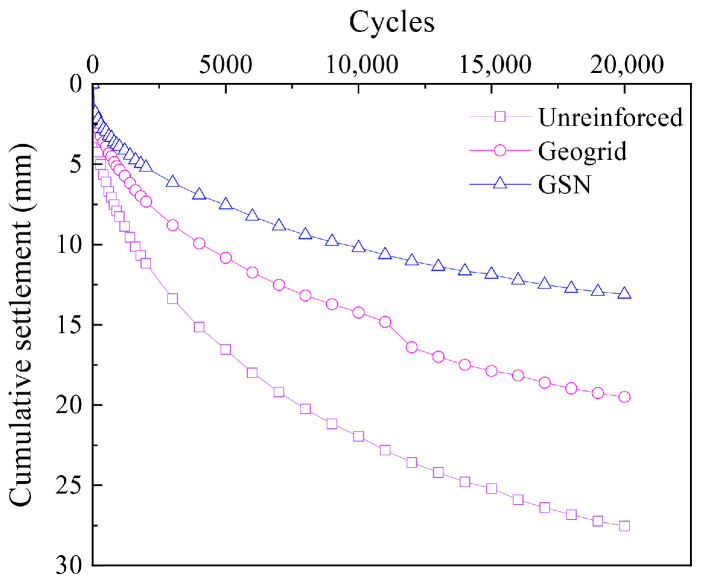
Variation of cumulative settlement with cycles for different cases.

For clarity, only representative cycle points were selected to plot the relationship between cumulative settlement and the number of cycles (Figure 8). In the early vibration stages, the settlements of all three conditions increased rapidly. Still, the geogrid-reinforced embankment effectively reduced the settlement, and the GSN-reinforced embankment showed even more significant reduction. As the number of cycles increased, the settlement rate slowed, and the reinforcement continued to limit settlement. After 20,000 cycles, the cumulative settlement at the center of the top surface of the unreinforced embankment was 27.55 mm. In contrast, the geogrid-reinforced embankment had a 19.5 mm settlement, a reduction of 29.22% compared to the unreinforced embankment. The GSN-reinforced embankment had a final settlement of 13.1 mm, a decrease of 32.82% compared to the geogrid-reinforced embankment and 52.45% compared to the unreinforced embankment.

#### 3.2.2. Cumulative Lateral Displacement

**Figure 9:**
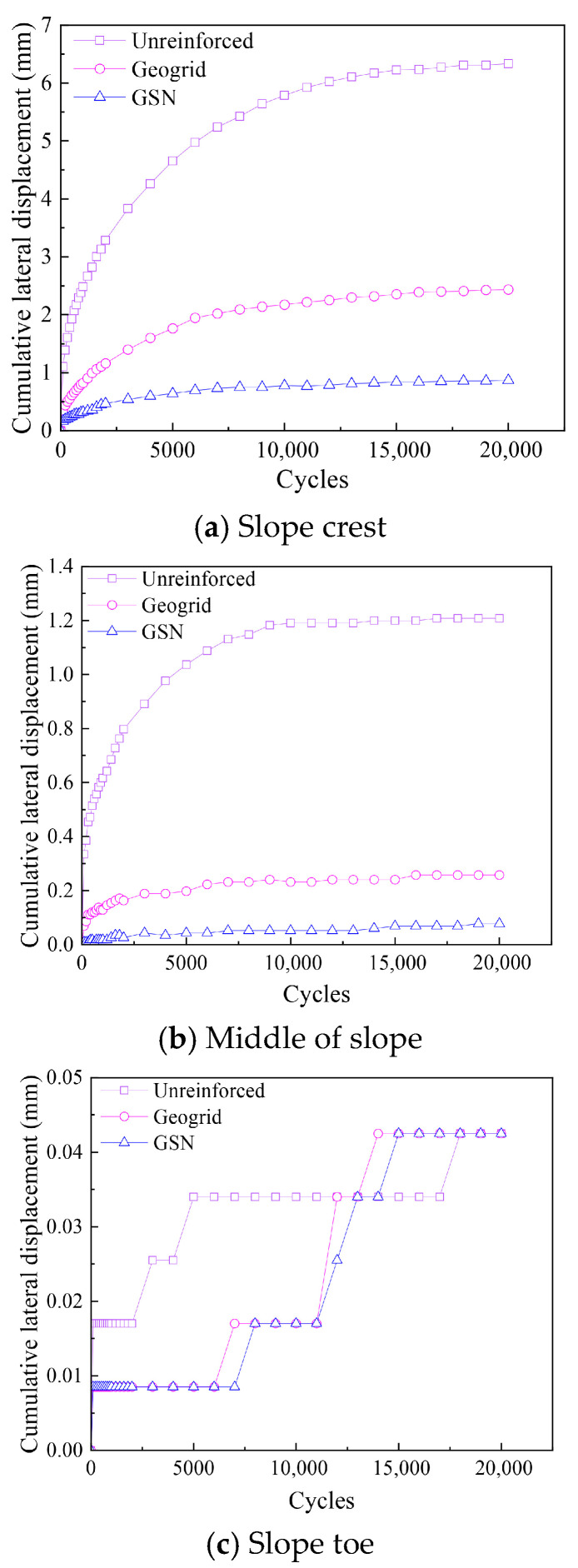
Cumulative lateral displacements at different positions of embankment slopes under various cases over cycles.

The cumulative lateral displacement of the slope for various conditions as a function of the number of cycles is shown in Figure 9. Evidently, at any given cycle, the lateral displacement decreased from the top of the slope to the toe. Under the same cycle, the geogrid-reinforced embankment effectively limited the lateral displacement at the top and middle of the slope. In contrast, the GSN-reinforced embankment showed an even more significant lateral displacement reduction than the geogrid. The displacement changes were less noticeable at the toe of the slope, and the differences among the three conditions were minimal. However, during the initial vibration stages, the reinforcement still effectively limited the toe displacement. After 20,000 cycles, the lateral displacement at the top of the slope for the unreinforced embankment was 6.34 mm. In contrast, the geogrid-reinforced embankment had a displacement of 2.44 mm, a reduction of 61.5% compared to the unreinforced embankment. The GSN-reinforced embankment had a lateral displacement of 0.87 mm at the top, a decrease of 64.34% compared to the geogrid-reinforced embankment and 86.28% compared to the unreinforced embankment, further limiting lateral displacement.

#### 3.2.3. Deformation Image Analysis

**Figure 10:**
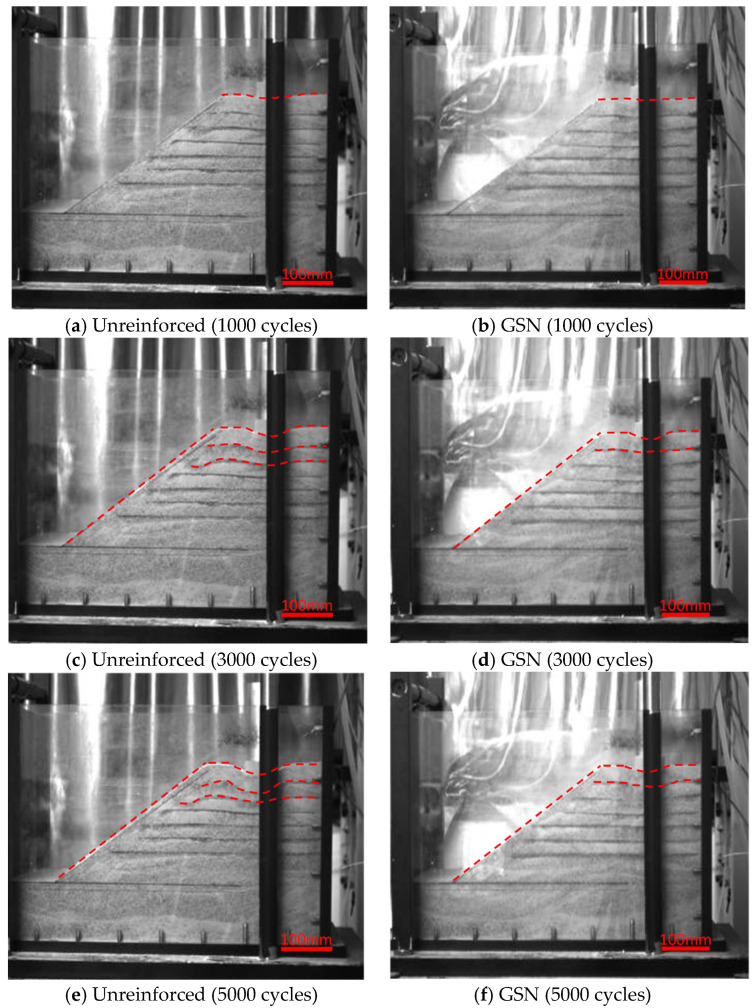
Deformation images of unreinforced and GSN-reinforced embankments under different cycles.

To better analyze the deformation of the embankment under cyclic loading, six layers of blackened sand were evenly distributed within 150 mm below the top of the embankment, and high-definition digital cameras were used to photograph the side profile of the embankment (Figure 10). The red dashed line in Figure 10 traces the deformation of the black sand and the outer contour of the slope. The images of the unreinforced embankment at different cycles (1000, 3000, 5000) are shown in Figure 10a,c,e. At 1000 cycles, the blackened sand layer directly below the loading plate moved downward, and there was slight bulging at the top and middle of the slope. As the number of cycles increased to 3000, the blackened sand layer below the loading plate bent downward, and the bulge at the top and middle of the slope became more pronounced. The blackened sand near the slope, which contained more fine sand, permeated downward. At 5000 cycles, the bulging at the top of the slope was significant, and the first two blackened sand layers below the loading plate were severely bent. However, the bottom three layers remained unchanged, indicating negligible lateral displacement at the toe, which aligns with the findings in Section 3.2.2.

The deformation process of the GSN-reinforced embankment is shown in Figure 10b,d,f. Due to the reinforcement effect, at the same number of cycles, both the settlement at the top and the lateral displacement on the side were significantly reduced compared to the unreinforced embankment. The primary changes were the downward bending of the first two blackened sand layers below the loading plate, but these changes were much less severe than in the unreinforced embankment.

#### 3.2.4. Dynamic Soil Pressure

**Figure 11:**
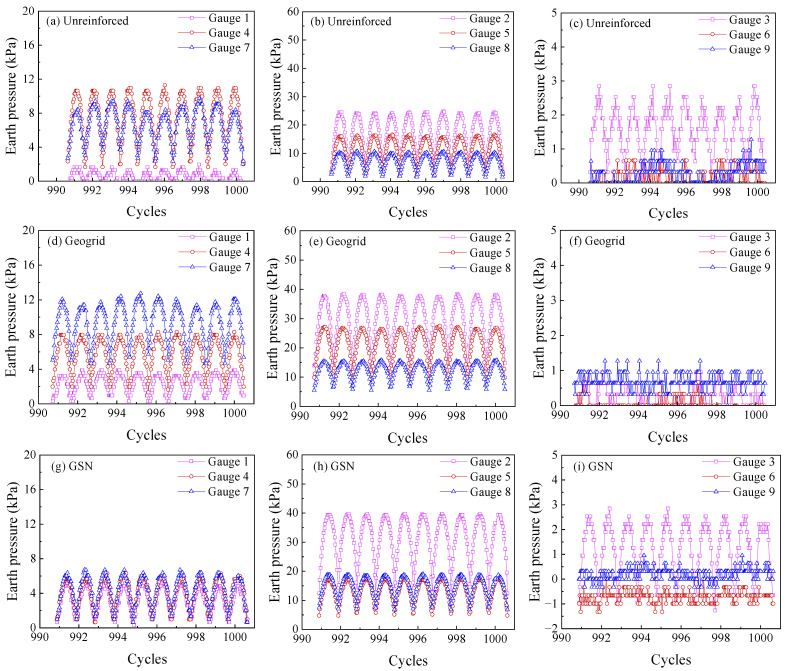
Dynamic earth pressure variations at different measuring points for unreinforced, geogrid-reinforced, and GSN-reinforced embankments: (**a**) unreinforced, gauge 1, 4, 7; (**b**) unreinforced gauge 2, 5, 8; (**c**) unreinforced, gauge 3, 6, 9; (**d**) geogrid, gauge 1, 4, 7; (**e**) geogrid, gauge 2, 5, 8; (**f**) geogrid, gauge 3, 6, 9; (**g**) GSN gauge 1, 4, 7; (**h**) GSN, gauge 2, 5, 8; (**i**) GSN, gauge 3, 6, 9.

Since the vibration lasted over 20,000 s, and the dynamic soil pressure had 20 data points per second, the data volume was extremely large. During the experiment, it was observed that after 1000 cycles, the soil pressure changes were relatively slow. Therefore, dynamic soil pressure values from around 1000 cycles were selected for analysis, as shown in Figure 11. The dynamic soil pressure exhibited a semi-sinusoidal waveform similar to the applied dynamic load. Comparing the soil pressure values at three measurement points below the loading plate (Points 2, 5, and 8 in Figure 11a–c), it was found that the peak soil pressure decreased from the top to the bottom of the embankment, as the additional stress was transmitted downward. The geogrid-reinforced embankment made the middle soil denser, and the soil pressure in the middle was significantly higher. The GSN-reinforced embankment showed even greater soil pressure in the middle, indicating that the soil was even denser, further enhancing the stiffness of the soil.

It is worth noting that the GSN-reinforced embankment increased the soil pressure at Point 8, indicating that the GSN reinforcement transferred the load to deeper soil layers, alleviating the local stress concentration in the unreinforced embankment and making the stress distribution more uniform [2]. From Figure 11d–f, it was observed that at the same horizontal level, the soil pressure values at Points 1, 4, 7 (on the left side of the embankment) and Points 3, 6, 9 (on the right side) were much lower than those at Points 2, 5, and 8 directly below the loading plate, consistent with the stress diffusion principle. The soil pressure values at Points 3, 6, and 9, closer to the slope, were within 3 kPa and could be neglected. The soil pressure at Points 1, 4, and 7 showed an increasing trend from top to bottom, which did not align with the stress diffusion principle. It was likely due to a strip load, and only half of the axially symmetric model was used. Points 1, 4, and 7 were close to the left side of the model box, which was a rigid plate, causing the lower soil to become denser, and the soil pressure to increase from top to bottom. Zhang et al. [7] conducted a 1:12 scale model test using a symmetrical half model to study reinforced embankments reinforced with horizontal–vertical inclusions under uniformly distributed loads. Their findings, similar to those in our study, revealed that near the side of the model box, the soil pressure, constrained by the steel plate, exhibited higher values in the lower layer compared to the upper layer. However, for the GSN-reinforced embankment, due to the significant reduction in soil settlement, the tendency of soil particles to move laterally was reduced, and the soil pressure values at Points 1, 4, and 7 were similar.

Combining the dynamic soil pressure distribution and embankment deformation analysis, it can be concluded that under the action of a strip load, the soil tends to move downward and laterally. The frictional and interlocking effects between the geogrid and the soil restrict lateral deformation, making the soil directly below the loading plate denser and significantly improving the embankment’s ability to resist shear stress. The strengthened nodes further limited soil movement through lateral resistance, forming a “reinforced zone” directly below the loading plate, resulting in higher soil pressure and greater load-bearing capacity.

## 4. Conclusions

This study evaluated the performance of GSN under static and dynamic strip loadings through laboratory model tests, revealing its significant advantages in enhancing embankment stability. The main conclusions are as follows:

(1) Compared to geogrid reinforcement, the ultimate bearing capacity of GSN-reinforced embankments increased by 26.2%, and the lateral displacement at the slope crest was reduced by 22.44%, demonstrating its superior performance under static loading.
(2) The GSN-reinforced embankment exhibited stronger fatigue resistance under dynamic loading. After 20,000 cyclic loadings, the cumulative settlement was reduced by 32.82%, and the lateral displacement at the slope crest was reduced by 64.34%, compared to geogrid reinforcement.
(3) Under dynamic loading, the soil pressure directly below the loading plate of GSN-reinforced embankments significantly increased and effectively transferred the load to deeper soil layers, significantly alleviating local stress concentration in unreinforced embankments and making the load distribution more uniform.
(4) By strengthening the nodes, GSN used lateral resistance to limit lateral deformation of the soil, forming a stable “reinforcement zone”. This optimized load transfer paths, significantly enhancing the overall stiffness and shear strength of the soil, thereby substantially improving the bearing capacity and stability of the embankment.

Although this study has made certain progress, there are still limitations in some aspects, such as the scale effects, the lack of long-term testing, variability in GSN construction, and simplifications in the loading. Future research will further validate the impact of scale effects, focusing on the loading simplification problem mentioned in the paper and refining actual loading conditions such as waveform, frequency, dynamic load amplitude, etc. Simultaneously, long-term tests will be conducted to evaluate the performance of structures under real-world operational conditions. Additionally, the impact of construction variability on structural performance will be studied, with optimization recommendations provided.

## Figures and Tables

**Table 1:** Basic parameters of the sand sample.

Property	Value
*D*_10_ (mm)	0.54
*D*_30_ (mm)	0.62
*D*_60_ (mm)	0.75
Specific gravity	2.64
Coefficient of uniformity	1.39
Coefficient of curvature	0.94
Minimum dry density (g/cm^3^)	1.58
Maximum dry density (g/cm^3^)	1.87

**Table 2:** Geogrid technical specifications.

Property	Value
Longitudinal/transverse mean tensile strength (kN/m)	25
Longitudinal/transverse tensile strength at 2% strain (kN/m)	12
Longitudinal/transverse tensile strength at 5% strain (kN/m)	20
Strain at ultimate tensile strength (%)	10
Aperture size (mm)	50 × 50

**Table 3:** Geomembrane technical specifications.

Property	Value
Density (g/cm^3^)	0.939
Tensile yield strength (N/mm)	22
Elongation at yield (%)	12
Tensile breaking strength (N/mm)	40
Elongation at break (%)	700

**Table 4:** Comparison of model dimensions from previous studies.

Reference	Model Dimensions (Length × Width × Height)	Model Length/Strip Footing Width	Model Height/Strip Footing Width
Tafreshi et al. (2010) [37]	750 mm × 150 mm × 375 mm	10	5
Mirzababaei et al. (2017) [38]	800 mm × 300 mm × 350 mm	16	7
Chen et al. (2021) [39]	800 mm × 200 mm × 480 mm	10	6
Zhang et al. (2024) [2]	600 mm × 290 mm × 333/343 mm	10	5.55~5.72
This study	600 mm × 290 mm × 300 mm	10	5

**Table 5:** Test cases.

Case	Reinforcement Layers	Reinforcement Material	Loading Form
1	-	Unreinforced	Static load
2	3	Geogrid	
3	3	GSN	
4	-	Unreinforced	Fixed amplitude dynamic load (20,000 cycles)
5	3	Geogrid	
6	3	GSN	

## Data Availability

Data are contained within the article.
